# The Magnitude of Varus Correction and Its Influence on Postoperative Patellar Height and Posterior Tibial Slope in Medial Open-Wedge High Tibial Osteotomy

**DOI:** 10.3390/jcm15041469

**Published:** 2026-02-13

**Authors:** Veljko Santic, Nikola Matejcic, Marta Santic, Nikola Grzalja

**Affiliations:** 1 Lovran Orthopedic Clinic, Faculty of Medicine, University of Rijeka, 51415 Lovran, Croatia; 2 Lovran Orthopedic Clinic, 51415 Lovran, Croatia

**Keywords:** opening wedge high tibial osteotomy, patellar height, patellar height measurements, patella infera, posterior tibial slope

## Abstract

**Objectives**: Medial open-wedge high tibial osteotomy (MOWHTO) is a surgical procedure used to treat medial compartment osteoarthritis of the knee with varus deformity. The aim of this study was to examine whether a larger correction angle in medial open wedge high tibial osteotomy (MOWHTO) leads to greater changes in postoperative patellar height (PH) and posterior tibial slope (PTS). **Methods**: Data from 83 patients who underwent MOWHTO were retrospectively analyzed. Lower limb alignment was evaluated using the hip–knee–ankle angle (HKAA). The PH was assessed using the Insall–Salvati index (ISI), the Blackburne–Peel index (BPI), the Caton–Deschamps index (CDI), the modified Miura–Kawamura index (MKI), and the plateau–patella angle (PPA). The PTS was determined using the Moore–Harvey method. **Results**: The median correction of HKAA was 8°. A decrease in PH was observed in the majority of cases across all methods, with the highest frequency of postoperative patella infera detected using the MKI. PTS most frequently increased, with a median increase of 3°. A significant association between the magnitude of HKAA correction and patellar height in the overall cohort was observed only for the MKI, whereas in patients with an HKAA correction ≥ 10°, significant associations were found for both the MKI and CDI. No significant association was found between the magnitude of HKAA correction and changes in posterior tibial slope in the overall cohort, while a significant negative correlation was observed in patients with an HKAA correction ≥ 10°. **Conclusions**: Assessment using the MKI demonstrated greater sensitivity in detecting postoperative PH decrease, particularly in identifying patella infera. The magnitude of HKAA correction was significantly associated with greater changes in PTS and PH in patients with a coronal plane correction of ≥10°.

## 1. Introduction

Medial open-wedge high tibial osteotomy (MOWHTO) is a surgical procedure used to treat patients with genu varum and medial tibiofemoral osteoarthritis. The aim of this procedure is to transfer load from the affected medial compartment to the healthy lateral compartment by changing the mechanical axis of the lower limb. This treatment is particularly useful in younger and physically active patients [1,2]. Numerous studies have reported that MOWHTO delays the need for knee arthroplasty by relieving pain and improving knee function [3]. The survival rate is reported as 92% at 10 years and 72% at 20 years after surgery in cases where the endpoint is total knee arthroplasty [4].

In addition to standard surgical complications, such as infection, fracture of the lateral cortex of the proximal tibia, and delayed bone union, several authors have reported that correction achieved by MOWHTO induces changes in the sagittal plane, affecting patellar height (PH) and posterior tibial slope (PTS), which may lead to unfavorable clinical outcomes [5,6]. A postoperative decrease in PH, with possible development of patella infera, leads to alterations in normal knee joint kinematics, increased patellofemoral contact pressure, and progression of patellofemoral osteoarthritis [7]. An increase in PTS may result in restricted knee extension, increased tensile load on the anterior cruciate ligament, and excessive anterior tibial translation in the sagittal plane, and contribute further to osteoarthritis progression [8]. In addition to accelerating the need for total knee arthroplasty, these changes may cause technical difficulties during knee replacement surgery, particularly regarding patellar eversion, correct positioning of the tibial component, and ligament balancing [9]. Consequently, numerous studies have focused on refining MOWHTO surgical techniques to avoid changes in proximal tibial morphology, thereby preserving normal knee biomechanics and, with appropriate patient selection, achieving optimal therapeutic outcomes [10].

The aim of this study was to assess the magnitude of varus correction and its influence on patellar height (PH) and posterior tibial slope (PTS), as well as the relationship between changes in PTS and PH in patients who underwent medial open-wedge high tibial osteotomy (MOWHTO) with insertion of a wedge-shaped cancellous bone allograft and plate fixation. Additionally, we aimed to compare the sensitivity of five different PH indices in the context of varying correction magnitudes. Our hypothesis was that larger varus correction angles would result in greater changes in PH and PTS.

## 2. Materials and Methods

### 2.1. Patients

We performed MOWHTO on 87 patients between 2016 and 2021. Four patients were excluded from the study, leaving a total of 83 patients. The study included 31 women and 52 men, with a median age of 51 years, ranging from 28 to 57 years old (average of 49.7 ± 6.1). All surgeries were performed by a team of experienced surgeons using the same surgical technique and under the same conditions. The inclusion criteria were preserved knee range of motion, radiographic evidence of varying grades of medial compartment osteoarthritis accompanied by medial joint pain, and varus malalignment with a hip–knee–ankle angle (HKAA) of less than 180°. Exclusion criteria included lateral compartment or patellofemoral osteoarthritis, associated anterior knee pain, rheumatoid or psoriatic arthritis, Kellgren–Lawrence grade 4 medial compartment osteoarthritis, knee flexion contracture greater than 10°, and a history of previous open-knee surgery [11]. The protocol of this retrospective study was approved by the institutional ethics committee.

### 2.2. Operative Technique and Postoperative Rehabilitation

**Figure 1:**
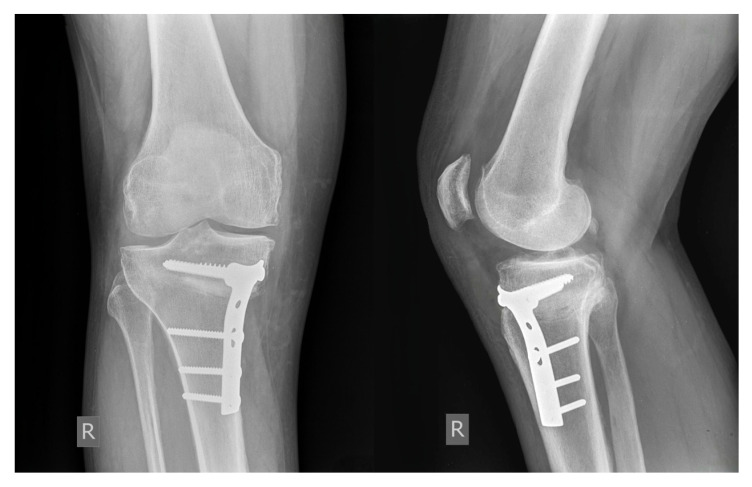
Anteroposterior and lateral radiographs after a MOWHTO using a cancellous bone allograft and fixation with a T-profile AO plate and screws (Shimadzu CH-200, Kyoto, Japan).

Standard single-plane MOWHTO was performed under tourniquet control and spinal anesthesia. The osteotomy site was previously marked under fluoroscopy using K-wires. A supra-tuberosity osteotomy was directed toward the fibular head, 3 to 4 cm distal to the medial tibial plateau in the coronal plane. In the sagittal plane, the osteotomy was performed above a Kirschner wire that had been intraoperatively positioned parallel to the tibial plateau under fluoroscopic control [12]. Particular care was taken when performing osteotomy at the posterior tibial cortex to avoid neurovascular injury. The osteotomy was slowly opened to achieve a gap size determined by preoperative digital planning. After this, a wedge-shaped bone allograft, with a medial base, was inserted posteriorly into the opened osteotomy site. The bone allografts were obtained from unfrozen femoral heads stored at −80 °C [13]. In order to preserve some degree of stability between bone fragments, it is important not to break the lateral cortex [14]. Internal fixation was achieved using a T-profile AO plate, as shown in Figure 1. Postoperatively, patients received low-molecular-weight heparin for thromboprophylaxis for a period of 6 weeks. The knee was not immobilized postoperatively, and patients were immediately enrolled in a supervised rehabilitation program to preserve muscle strength and prevent patella infera or arthrofibrosis [15]. Partial weight bearing (50%) was allowed after 6 weeks, followed by gradual increases on a weekly basis. Full weight bearing was permitted from week 12 onward, depending on radiographic findings.

### 2.3. Clinical and Radiographic Assessment

Following clinical examination, digital radiographic evaluation was performed preoperatively and postoperatively for each patient (IMPAX, Agfa). Anteroposterior radiographs were obtained to assess medial compartment osteoarthritis using the Kellgren–Lawrence scale; lateral knee radiographs at 30° of flexion were obtained to assess patellar height (PH) and posterior tibial slope (PTS) using a goniometer. To determine lower limb alignment, HKAA was measured on the standing long-leg anteroposterior radiographs, and its value was used in preoperative planning to determine the correction angle [16]. Patellar height was measured using five different methods: the Insall–Salvati index (ISI), the Blackburne–Peel index (BPI), the Caton–Deschamps index (CDI), the modified Miura–Kawamura index (MKI), and the plateau–patella angle (PPA) [17,18,19,20,21]. The PTS was measured as the angle between the tibial plateau and a line perpendicular to the anterior tibial cortex [22]. Radiographic measurements were independently performed by three experienced orthopedic surgeons before surgery and repeated 12 to 18 months postoperatively, at the time of implant removal. Implant removal was performed in patients to reduce local irritation and to facilitate potential future knee arthroplasty if required.

### 2.4. Statistical Analysis

Statistical analysis was performed using JASP statistical software (version 0.17.2.1). Continuous variables are presented as the median (range) and mean ± standard deviation. Median values are reported to account for potential non-normal distributions, while mean ± standard deviation is additionally provided for completeness and comparability with the existing literature. Categorical variables are presented as absolute numbers and percentages. Comparisons between preoperative and postoperative radiographic measurements were performed using paired-samples *t*-tests for normally distributed variables and the Wilcoxon signed-rank test for variables that did not meet normality assumptions. Relationships between changes in PH, PTS, and the magnitude of coronal plane correction expressed by the HKAA were evaluated using Spearman’s rank correlation coefficient (ρ). Correlation analyses were conducted for the entire cohort and for a predefined subgroup of patients with a correction angle ≥ 10°. Although interobserver reliability statistics were not calculated separately, the use of averaged measurements from three experienced surgeons was intended to minimize individual measurement bias. All statistical tests were two-tailed, and a *p*-value < 0.05 was considered statistically significant.

## 3. Results

Preoperative and postoperative PH measurements obtained using five different measurement methods are summarized in Table 1. All applied methods consistently demonstrated a postoperative decrease in PH at 12 to 18 months of follow-up. Although the magnitude of change varied among measurement techniques, a reduction in PH was observed in the majority of cases across all methods, and unchanged PH was recorded in a smaller proportion of knees. The incidence of postoperative patella infera differed depending on the measurement method, with the highest frequency detected using the MKI.

**Figure 2:**
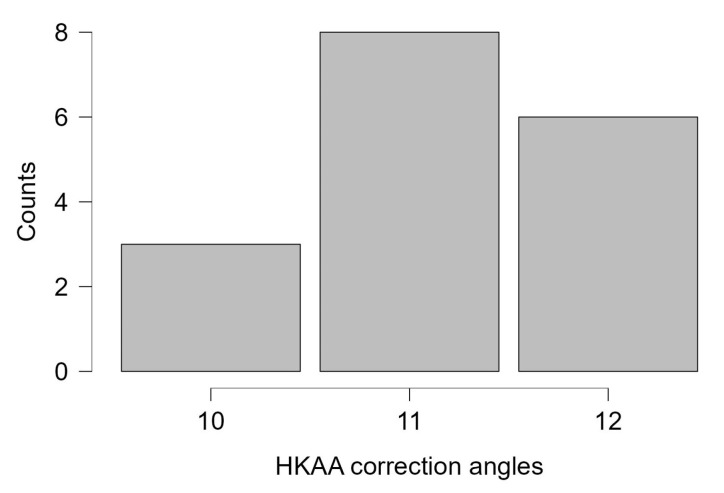
Distribution of HKAA correction angles in the subgroup with correction angle ≥ 10°.

Changes in PTS and HKAA following MOWHTO are presented in Table 2. The magnitude of coronal plane correction ranged from 6° to 12°, with a median correction of 8°. Postoperatively, PTS most frequently increased, with a median increase of 3°, while a smaller number of patients demonstrated no change in PTS. Seventeen patients had an HKAA correction angle ≥ 10°, and distribution of them is shown in Figure 2. Both PTS and HKAA showed statistically significant postoperative changes.

No statistically significant association was found between changes in PTS and the magnitude of HKAA correction in the overall cohort (ρ = 0.142; *p* = 0.199). In contrast, a significant negative correlation was observed among patients with an HKAA correction ≥ 10° between changes in PTS and HKAA correction (ρ = −0.522; *p* = 0.032).

The influence of changes in posterior tibial slope (PTS) and HKAA correction on postoperative patellar height is summarized in Table 3. In the overall cohort, significant correlations between changes in PTS and patellar height were observed for the BPI and PPA, whereas no significant associations were found for the CDI and ISI. When analyzing the relationship between the magnitude of HKAA correction and patellar height in the overall cohort, a significant association was identified only for the MKI. In contrast, in patients with an HKAA correction ≥ 10°, significant associations between HKAA correction and patellar height were observed for both the MKI and CDI.

## 4. Discussion

MOWHTO is a well-established joint-preserving treatment option for medial compartment knee osteoarthritis. Although the procedure is designed to restore coronal alignment while preserving normal knee biomechanics, numerous clinical and biomechanical studies have reported postoperative alterations in PH and PTS, in some cases contrary to mechanical expectations [23,24,25,26]. Furthermore, variability in reported changes in PH has been observed even among studies investigating identical high tibial osteotomy techniques, highlighting inconsistencies related to measurement methods and study design [15,27,28]. Surgical accuracy in osteotomy surgery is contingent on multiple factors including the measurement used for the planned correction. Recent advances in three-dimensional (3D) preoperative planning have been introduced in high tibial osteotomy to improve the accuracy of coronal and sagittal alignment correction [10,29,30].

Currently, many surgeons favour biplanar MOWHTO techniques, which have been reported to provide greater stability and may reduce postoperative changes in patellar height. In the present study, we used a single-plane technique with posterior wedge positioning; therefore, the observed changes in patellar height (PH) and posterior tibial slope (PTS) may not be directly applicable to biplanar osteotomies. Future studies comparing sagittal plane changes between mono and biplanar techniques would be valuable [10].

Because no single method exists to assess PH as a gold standard, five commonly used measurement techniques were applied in the present study to obtain a comprehensive and objective assessment. These methods were selected acknowledging that each has specific limitations and that a certain degree of measurement variability is inherent to all techniques. The ISI, BPI, CDI, and MKI indices are based on ratios of defined anatomical lengths, whereas the PPA represents a direct angular measurement expressed in degrees. In contrast to tibia-based indices, the MKI determines PH relative to a femoral reference point and is, therefore, not affected by geometric changes in the proximal tibia following osteotomy. It should also be considered that the ISI reflects patellar tendon length, which may be affected by postoperative tendon shortening; similarly, quadricep hypotrophy may influence measurements obtained using the MKI [23,26]. The use of a wedge-shaped cancellous bone allograft during MOWHTO was intended to preserve the sagittal plane orientation of the osteotomy in addition to achieving coronal plane correction. Consequently, the role of the fixation device was limited to maintaining the achieved osteotomy height and alignment [31]. Radiographic follow-up measurements were performed at the time of implant removal, when bone healing and remodeling were expected to be complete and when potential postoperative changes in ligamentous structures that could influence PH or PTS were considered stabilized.

Measurements of PH obtained using the ISI, BPI, CDI, PPA, and MKI demonstrated that postoperative PH remained unchanged in only a small proportion of patients; by contrast, a decrease in PH was observed in the majority of cases. The incidence of postoperative patella infera varied among measurement methods, with the highest frequency detected using the MKI, which identified patella infera in 30.1% of patients, similar to previous reports by LaPrade et al. [23]. These results led us to question the sensitivity of patellar height measurements using the ISI, BPI, CDI, and PPA after MOWHTO, which are influenced by geometric changes in the proximal tibia. By contrast, the MKI has been shown to be methodologically more stable and clinically sensitive, as it uses femoral reference points [32].

Changes in the PTS may substantially affect knee function due to its important role in knee biomechanics. In the present study, postoperative PTS values increased in the majority of patients, with median values rising from 11° preoperatively to 14° at follow-up. An increase was observed in 82% of cases. These findings are consistent with previous reports indicating that MOWHTO may lead to increased PTS and a concomitant reduction in PH [27,28]. In contrast to cadaveric studies by Ruzbarsky et al. and clinical investigations by Mabrouk et al., which demonstrated that PTS can be preserved by optimizing intraoperative techniques, only 15 patients (18%) in the present study showed no postoperative change in PTS after the surgical technique was applied [33,34]. A reason for the variability in reported changes in PTS and PH following MOWHTO could be that all measurements were obtained from plain radiographs. Namely, plain radiographs have inherent limitations in terms of reproducibility. The use of CT scans in the analysis of these radiological parameters, due to their higher precision and the possibility of three-dimensional analysis of the proximal tibia after MOWHTO, could lead to more reliable results [32,35].

When examining the relationship between changes in PH and PTS, significant associations were observed for the BPI and PPA; however, no statistically significant association was identified using the CDI, despite a tendency toward decreased patellar height. In contrast, there was no association between changes in PTS and PH when assessed using the ISI. The findings obtained with the BPI, PPA, and ISI are generally consistent with previously published studies [28]. The lack of statistical significance observed with the CDI was partially unexpected, as this measurement is theoretically sensitive to geometric alterations of the proximal tibia following MOWHTO. The absence of an association between PH and PTS when assessed using the ISI may be related to the surgical technique and postoperative rehabilitation protocol, suggesting possible limited alterations in patellar tendon length [22].

Changes in PH and PTS are generally considered to be related to coronal plane correction during MOWHTO. Accordingly, the aim of the present study was to evaluate the influence that the magnitude of HKAA correction has on these radiographic parameters. In the analysis of the entire cohort, no statistically significant correlations were identified between the magnitude of HKAA correction and a decrease in PH when assessed using the ISI, BPI, CDI, and PPA. These findings suggest that, in most cases, the degree of HKAA correction does not have a direct measurable effect on postoperative PH, which is consistent with the results reported by El Amrani et al. [28]. By contrast, we observed a moderate association between HKAA correction and a decrease in PH when assessed using the MKI. Furthermore, no statistically significant relationship was found between the magnitude of HKAA correction and changes in PTS in the overall cohort. Given that previous studies have identified an HKAA correction of ≥10° as a potential predictor of patellofemoral osteoarthritic changes, a separate analysis was performed in this subgroup of patients [36]. As a result, significant associations were observed between HKAA correction and PH for both the MKI and CDI. Additionally, a significant association was identified between the magnitude of HKAA correction and the increase in PTS. However, these findings should be interpreted with caution, as this subgroup comprised a relatively small number of patients (17 cases, approximately 20% of the total cohort).

Taken together, these results suggest that the extent of radiographic changes in PH and PTS following MOWHTO cannot be reliably predicted based solely on the magnitude of coronal plane correction. A clearer pattern of association was observed primarily in patients with preoperative low patellar height, who are at risk of developing patella infera. This was also true for cases involving larger varus corrections (≥10°), where significant associations were identified between PH decrease and increases in PTS. Therefore, in patients presenting with natural patella infera, careful preoperative planning is recommended when performing MOWHTO, particularly in cases requiring larger coronal plane corrections. Surgeons should consider surgical techniques that reduce the risk of further patellar height reduction, such as distal tibial tubercle osteotomy.

A limitation of the present study is the absence of clinical outcome measures. Although significant radiographic changes in PH and PTS were observed, the direct clinical relevance of these findings cannot be determined from the present data. Therefore, the results should primarily be interpreted as biomechanical and radiographic observations, and future studies should integrate clinical outcome measures to determine the functional impact of these changes.

## 5. Conclusions

Although all analyzed measurement methods consistently detected a postoperative decrease in pH following MOWHTO, their sensitivity differed. The assessment using the MKI demonstrated greater sensitivity in detecting postoperative decreases in PH, particularly patella infera. The hypothesis that larger varus correction is associated with greater changes in PTS and PH was only partially supported, with evidence primarily found in patients with a coronal plane correction of ≥10°.

## Figures and Tables

**Table 1:** Patellar height according to different measurement methods before surgery and 12 to 18 months postoperatively.

Methods of PH Measurement (Normal Range)	Parameters	Preoperative	12–18 Months Postoperative	*p*-Value *
ISI (0.8–1.2)	Average measurement	0.98 (0.81–1.31) 1 ± 0.12	0.94 (0.69–1.31) 0.95 ± 0.12	<0.001
	Knees with patella decrease/patella infera (ISI < 0.8)		74 (89%)/4 (4.8%)	
	Knees without change in PH		9 (10.8%)	
BPI (0.54–1.06)	Average measurement	0.97 (0.68–1.25) 0.97 ± 0.13	0.88 (0.51–1.21) 0.88 ± 0.13	<0.001
	Knees with patella decrease/patella infera (BPI < 0.54)		74 (89%)/1 (1.2%)	
	Knees without change in PH		9 (10.8%)	
CDI (0.6–1.3)	Average measurement	1.05 (0.71–1.39) 1.05 ± 0.15	0.96 (0.59–1.31) 0.95 ± 0.14	<0.001
	Knees with patella decrease/patella infera (CDI < 0.6)		79 (95.2%)/2 (2.4%)	
	Knees without change in PH		4 (4.8%)	
PPA (21–29°)	Average measurement	27.6 (21.7–32.9) 27.28 ± 2.35	25.1 (19.8–32.4) 25 ± 2.96	<0.001
	Knees with patella decrease/patella infera (PPA < 21°)		72 (86.7%)/6 (7.2%)	
	Knees without change in PH		11 (13.2%)	
MKI	Average measurement	1.01 (0.65–1.38) 1 ± 0.13	0.95 (0.54–1.24) 0.94 ± 0.13	<0.001
	Knees with patella decrease/patella infera (change > 10%)		70 (84.3%)/25 (30.1%)	
	Knees without change in PH		13 (15.7%)	

* *p*-values are reported only for paired comparisons of preoperative and postoperative continuous variables. Counts of knees with decreased PH, patella infera, or unchanged PH are presented descriptively.

**Table 2:** Changes in PTS and HKAA correction from preoperative to the 12- to 18-month follow-up.

Parameters	Preoperative	12–18 Months Postoperative	*p*-Value *
	Average Measurement		
PTS angle	11° (3–20.7) 11.53° ± 3.27	14° (6–22) 13.94° ± 3.42	<0.001
Patients with increased PTS angle		68 (82%)	
Patients without change in PTS angle		15 (18%)	
HKAA correction	177° (174–178) 176.73° ± 1.18	185° (182–188) 184.72° ± 1.67	<0.001
Patients with HKAA correction < 10°		66 (79.5%)	
Patients with HKAA correction ≥ 10°		17 (20.5%)	

* *p*-values are reported only for paired comparisons of preoperative and postoperative continuous variables. The number and percentage of patients with increased PTS, without change in PTS, with HKAA correction < 10°, and with HKAA correction ≥ 10° are presented descriptively.

**Table 3:** Presentation of the results of the relationship between PTS and PH, and between HKAA correction and PH, for the whole group of patients and specifically for patients with an HKAA correction ≥ 10°.

Methods of PH Measurement	PTS Angle		HKAA Correction	
	Spearman ρ	*p*-Value	Spearman ρ	*p*-Value
ISI	0.040	0.720	−0.013	0.905
BPI	0.350	0.001	0.207	0.060
CDI	0.170	0.123	0.046	0.680
PPA	0.438	<0.001	0.123	0.270
MKI			0.325	0.028
Group of 17 patients with HKAA correction ≥ 10°				
ISI	−0.226	0.383	0.123	0.639
BPI	0.465	0.060	−0.199	0.444
CDI	−0.390	0.122	0.679	0.003
PPA	0.532	0.028	−0.447	0.072
MKI			0.649	0.005

## Data Availability

The original contributions presented in this study are included in the article. Further inquiries can be directed to the corresponding author.

## Abbreviations

The following abbreviations are used in this manuscript:

MOWHTO	Medial open-wedge high tibial osteotomy
PH	Patellar height
PTS	Posterior tibial slope
HKAA	Hip–knee–ankle angle
ISI	Insall–Salvati index
BPI	Blackburne–Peel index
CDI	Caton–Deschamps index
MKI	Modified Miura–Kawamura index
PPA	Plateau–patella angle
